# E47 and Id1 Interplay in Epithelial-Mesenchymal Transition

**DOI:** 10.1371/journal.pone.0059948

**Published:** 2013-03-26

**Authors:** Eva Cubillo, Antonio Diaz-Lopez, Eva P. Cuevas, Gema Moreno-Bueno, Hector Peinado, Amalia Montes, Vanesa Santos, Francisco Portillo, Amparo Cano

**Affiliations:** 1 Departamento de Bioquímica, Facultad de Medicina, Universidad Autónoma de Madrid (UAM), Instituto de Investigaciones Biomédicas “Alberto Sols” (CSIC-UAM), IdiPAZ, Madrid, Spain; 2 MD Anderson Cancer Center Madrid, Madrid, Spain; Bellvitge Biomedical Research Institute (IDIBELL), Spain

## Abstract

E12/E47 proteins (encoded by *E2A* gene) are members of the class I basic helix-loop-helix (bHLH) transcription factors (also known as E proteins). E47 has been described as repressor of *E-cadherin* and inducer of epithelial-mesenchymal transition (EMT). We reported previously that EMT mediated by E47 in MDCK cells occurs with a concomitant overexpression of Id1 and Id3 proteins. Id proteins belong to class V of HLH factors that lack the basic domain; they dimerise with E proteins and prevent their DNA interaction, thus, acting as dominant negative of E proteins. Here, we show that E47 interacts with Id1 in E47 overexpressing MDCK cells that underwent a full EMT as well as in mesenchymal breast carcinoma and melanoma cell lines. By conducting chromatin immunoprecipitation assays we demonstrate that E47 binds directly to the endogenous *E-cadherin* promoter of mesenchymal MDCK-E47 cells in a complex devoid of Id1. Importantly, our data suggest that both E47 and Id1 are required to maintain the mesenchymal phenotype of MDCK-E47 cells. These data support the collaboration between E47 and Id1 in the maintenance of EMT by mechanisms independent of the dominant negative action of Id1 on E47 binding to *E-cadherin* promoter. Finally, the analysis of several N0 breast tumour series indicates that the expression of *E47* and *ID1* is significantly associated with the basal-like phenotype supporting the biological significance of the present findings.

## Introduction

Epithelial-mesenchymal transition (EMT) is presently recognised as a key process for tumour invasion and metastasis [1]–[3]. The hallmarks defining the EMT process are the loss of E-cadherin mediated cell-cell adhesion and epithelial cell polarity concomitant to the acquisition of mesenchymal markers and increased motility and invasiveness [2]–[4]. Several transcriptional factors, presently called EMT-TFs, have been identified as EMT inducers, many of them also acting as direct *E-cadherin* repressors. Among them, factors from the Snail (Snail1/Snail2), Zeb (ZEB1/ZEB2) and bHLH (E47, Twist1, E2-2) families have been described [1], [2], [5].

The molecular mechanisms underlying the action of the various EMT-TFs are unequally known for Snail1 [6]–[11], Snail2 and ZEB1 factors [12], [13]. Much less is known on the mechanism of bHLH factors participating in EMT [14].

Class I bHLH E12/E47 are two splice variants of the *E2A* (also known as *TCF3*) gene product. E2A proteins regulate gene target expression as homodimers or heterodimers with other bHLH factors [15]. Id proteins (Id1-4), which lack the basic DNA binding domain, associate with E2A proteins and prevent them from binding to DNA [16]. A role for Id1 in mediating EMT-induced by TGFbeta or other factors has been proposed. The action of Id1 in EMT appears to depend on its dominant negative action on E2A factors in some systems [17], [18], but mediated by interaction with cytosolic/membrane proteins in other situations [19]. Interestingly, Id1 expression in breast cancer cells has been correlated with poor prognosis and lung metastasis [20], [21], [22]. Moreover, mice lacking Id1/Id3 genes are resistant to tumour neoangiogenesis and tumour cells failed to grow and/or metastasize [23], [24]. More recently, Id1 has been shown to be required for self-renewal of adult neural stem cells and glioblastoma tumours [25], [26] and for the mobilization of endothelial precursor cells to lung metastasis [27], further reinforcing the relevance of Id1 in tumorigenesis.

Our previous studies demonstrated that E47 is a strong EMT inducer and *E-cadherin* repressor [28], [29], but the specific mechanisms mediating E47 actions and whether E47 expression is required for EMT induction and/or maintenance of the mesenchymal phenotype are still unknown. Our previous analysis showed that Id factors, particularly Id1 and Id3, are strongly upregulated in MDCK cells stably expressing E47 or E2-2 factors [30]–[32]. The present study analyse the interplay between E47 and Id1 in *E-cadherin* repression and EMT. Our results reveal that E47 mediates *E-cadherin* transcriptional repression by direct interaction with its promoter in a complex devoid of Id1. Sustained expression of E47 and Id1 is required to maintain the mesenchymal phenotype of MDCK-E47 cells and to preserve cell viability. Remarkably, *E47* as well as *ID1* mRNAs are more frequently expressed in basal-like breast carcinomas compared to non-basal tumours supporting the participation of these proteins in defining this aggressive breast tumour subtype.

## Results

### Id1 protein is upregulated and interacts with E47 in MDCK-EGFP-E47 cells

**Figure 1:**
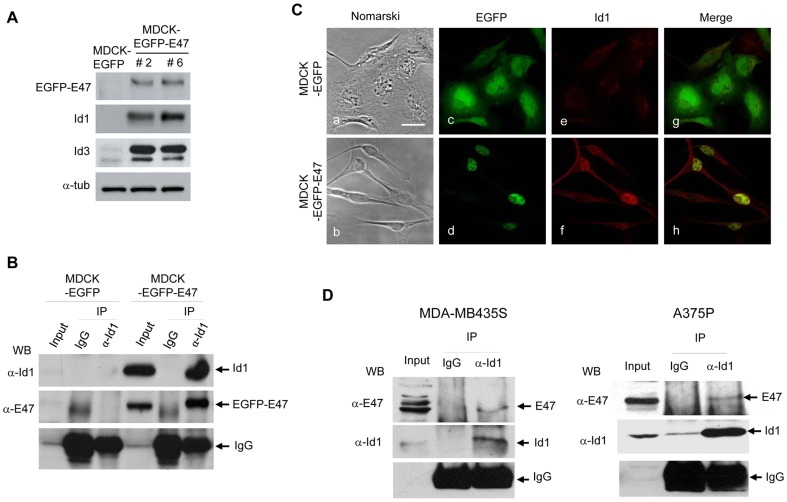
Id1 is upregulated and interacts *in vivo* with E47 in MDCK-EGFP-E47 cells. (**A**) Cell extracts derived from MDCK-EGFP and MDCK-EGFP-E47 cells were analysed for expression of EGFP-E47, Id1 and Id3 proteins by Western blot; alpha-tubulin was used as loading control. Two independent clones (#2, #6) generated after stable expression of EGFP-E47 in MDCK cells are shown. (**B**) Cell extracts derived from MDCK-EGFP and MDCK-EGFP-E47 cells were immunoprecipitated with anti-Id1 antibodies and control IgGs and analysed by Western blotting with the indicated anti-Id1 and anti-E2A antibodies. (**C**) Nomarski (a,b) and confocal immunofluorescence images (c-h) of MDCK-EGFP and MDCK-EGFP-E47 cells. Localization of EGFP (c,g), EGFP-E47 (d,h) and Id1 (e,f) is shown. See the colocalization of Id1 with EGFP-E47 in the nuclei (merged image, h). Bars, 25 um. (**D**) Cell extracts derived from MDA-MB435S (left panels) and A375P (right panels) cells were immunoprecipitated with anti-Id1 antibodies and control IgGs and analysed by Western blotting with the indicated anti-Id1 and anti-E2A antibodies.

To further characterize the E47/Ids interplay during EMT, and because of the lack of reliable anti-E47 commercial antibodies for immunoprecipitation assays, we generated stable transfectant MDCK-EGFP-E47 cells. Extensive characterization of MDCK-EGFP-E47 cells was performed in three independent clones that showed almost complete repression of E-cadherin and the same mesenchymal phenotype and properties than previously described for MDCK-E47 cells including a full EMT conversion and overexpression of Id1 and Id3 factors (Figure 1A; Figure S1).

To confirm the interaction between EGFP-E47 and Id proteins, co-immunoprecipitation analyses were performed. EGFP-E47 protein strongly interacts with Id1 (Figure 1B) in MDCK-EGFP-E47 cells. Confocal analysis in MDCK-EGFP-E47 cells showed that Id1 protein was localized mainly in the nuclei; Id1 co-localized with EGFP-E47 in the majority of cells (Figure1C, panels f, h). Co-immunoprecitation analysis with Id3 showed a much weaker interaction with EGFP-E47 compared to Id1 (data not shown). Therefore, we focus our following studies on Id1. To confirm E47-Id1 interaction, co-immunoprecipitation assays were also performed in mesenchymal breast carcinoma MDA-MB435S and melanoma A375P cells, previously shown to be E-cadherin deficient and to overexpress E47 and Id1 factors ([29], [31] and data not shown). Results obtained indicated the interaction between E47 and Id1 both cell lines (Figure 1D) thus confirming our observations in MDCK-E47 cells.

### Id1 is unable to overcome *E-cadherin* repression in MDCK-E47 cells

**Figure 2:**
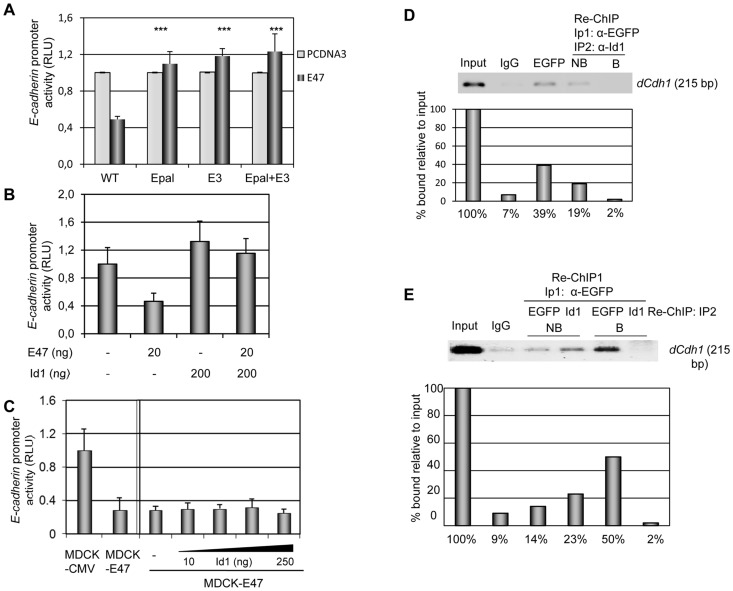
E47 represses *E-cadherin* by direct binding to the endogenous promoter in complexes devoid of Id1. (**A**) Dependence of E47 repression of *E-cadherin* promoter on proximal E-boxes. HEK293T cells transiently co-transfected with 200 ng of the proximal wild type mouse E-cadherin-Luciferase promoter (−178/+92 bp) or point mutants in the proximal E-boxes (E-pal +/− E-box3) and 10 ng of CMV-beta-gal plasmid in the presence of 50 ng of pcDNA3-E47 (dark grey bars) or control pcDNA3 (light grey bars) vectors, as indicated. Differences between the E47 mediated repression of the mutants versus the wild type promoter were statistically analysed by t-Student test (***p<0.001). (**B**) and (**C**) *E-cadherin* promoter activity in MDCK (**B**) and MDCK-CMV and MDCK-E47 (**C**) cells transiently co-transfected with 200 ng of the proximal mouse E-cadherin-Luciferase promoter (−178/+92 bp) and 20 ng of TK-Renilla (**B**) or 10 ng of CMV-beta-gal (**C**) plasmids, in combination with the indicated amounts of pcDNA3-E47 and/or pcDNA3-Id1 vectors. RLU is normalized to that detected with empty pcDNA3 vector (**A, B**) or in control MDCK-CMV cells (**C**, left); results represent the mean of at least two independent experiments +/− s.d. (**D**) and (**E**) Chromatin immunoprecipitation (ChIP) assays of EGFP-E47 at the endogenous *E-cadherin* promoter in MDCK-EGFP-E47 cells. (**D**) ChIP assays were first performed with an antibody specific for EGFP or control IgG and the bound fraction analysed for endogenous *E-cadherin* promoter sequences (*dCdh1*) (left three lanes), followed by re-ChIP with anti-Id1 antibodies (IP2) and analyses of the bound (B) and unbound (NB) fractions for *E-cadherin* promoter sequences (*dCdh1*) (right two lanes). The input fraction (1/100) is shown at the left. Quantification of the amplified endogenous *E-cadherin* promoter compared to the input fraction (1/100) is shown in the lower panel. (**E**) ChIP assays were first performed with anti-EGFP (IP1) followed by re-ChIP (IP2) with anti-EGFP or anti-Id1 antibodies, as indicated, followed by analyses of the bound (B) and unbound (NB) fractions for *E-cadherin* promoter sequences (*dCdh1*). Input (1/100) and control IgG ChIP are shown on the left two lanes. Quantification of the amplified endogenous *E-cadherin* promoter compared to the input fraction (1/100) is shown in the lower panel.

Since Id proteins have been extensively described as dominant negative of DNA binding of E47 and other class I bHLH factors [15], [16], we then analysed their effect on E47 repression of the *E-cadherin* promoter. We first confirmed that E47 repression depends on the proximal E-boxes, E-pal and E3, of the mouse *E-cadherin* promoter (Figure 2A), in agreement with previous observations on the dependence of the E-pal element for *in vitro* binding of E47 [28]–[33]. The E47-mediated repression of *E-cadherin* promoter in MDCK cells was fully overcome by transient co-transfection with Id1 (Figure 2B). We then analysed the effect of Id1 proteins in MDCK cells stably expressing E47 with a complete EMT and exhibiting very low *E-cadherin* promoter activity [29]. Transient Id1 overexpression was unable to de-repress *E-cadherin* promoter activity in MDCK-E47 cells (Figure 2C). Similar results were obtained in MDCK-EGFP-E47 cells (unpublished data). Moreover, Id1 overexpression did not de-repress the basal activity of *E-cadherin* promoter in MD-MDA435S and A375P cells (Figure S2). Altogether, these results demonstrate that *E-cadherin* repression by E47 is not sensitive to inhibition by Id1 protein once a full EMT is achieved.

### E47 interacts *in vivo* with the *E-cadherin* promoter in complexes devoid of Id1

The interaction of E47 with the endogenous *E-cadherin* promoter has not yet been studied at the chromatin level. To assess this point, we performed chromatin immunoprecipitation (ChIP) assays in MDCK-EGFP-E47 cells with anti-EGFP antibodies. EGFP-E47 factor binds specifically to the endogenous *E-cadherin* promoter in MDCK-EGFP-E47 cells compared to unspecific IgG control (Figure 2D). To further characterize the E47 complexes bound to the *E-cadherin* promoter, re-ChIP assays were carried out. A sequential immunoprecipitation with anti-EGFP followed by anti-Id1 antibodies showed the absence of Id1 binding to the *E-cadherin* promoter in the chromatin fraction bound by EGFP-E47 (Figure 2D, re-ChIP, B fraction), while the unbound fraction from the Id1 precipitates still retain a significant part of the endogenous *E-cadherin* promoter (Figure 2D, re-ChIP NB fraction). The specificity of the re-ChIP assay was further confirmed by parallel re-ChIP assays with anti-EGFP and anti-Id1 antibodies (Figure 2E) that showed the ability of anti-EGFP to effectively precipitate the remaining *E-cadherin* promoter from the Id1-ChIP fraction and the complete absence of Id1 binding to the EGFP-E47 bound fraction (Figure 2E, two most right lanes). These results indicate that E47 interacts *in vivo* directly with the endogenous *E-cadherin* promoter in MDCK-EGFP-E47 cells in complexes devoid of Id1.

### Sustained E47 expression is essential for the maintenance of EMT and tumour aggressiveness

**Figure 3:**
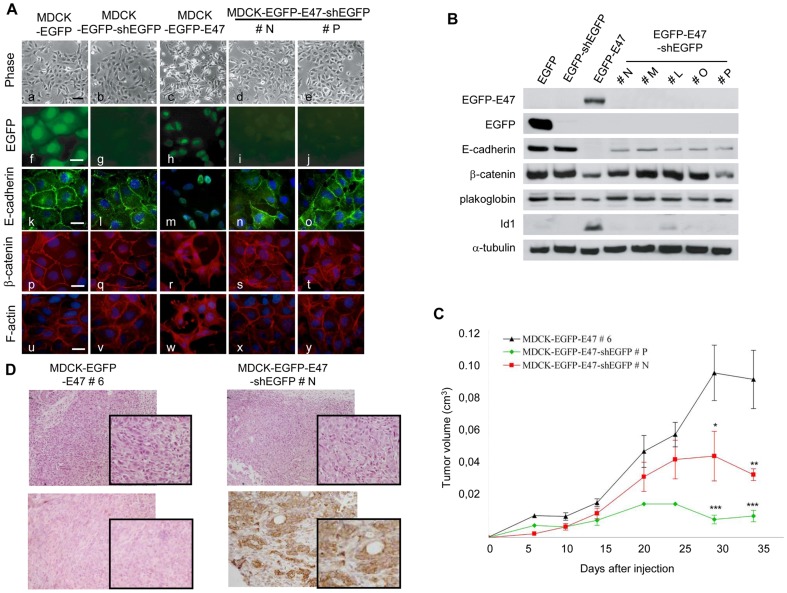
E47-silencing in MDCK-EGFP-E47 cells induces a MET process, downregulation of Id1 and reduction of tumour growth. (**A**) Phase contrast images (a-e) and immunofluorescence images (f–y) of the indicated cell lines showing localization of EGFP (f,g), EGFP-E47 (h–j), Ecadherin (k–o), beta-catenin (p–t) and F-actin organization (u–y). Nuclei were stained with DAPI (k–y). Black bar, 60 um (a–e); white bars, 25 um (f–y). (**B**) Western blot analyses of epithelial markers and Id1 in the indicated cell lines; alpha-tubulin was used as loading control. Two (#N, #P) and five independent clones (#N, #M, #L, #O, #P) generated after stable expression of shEGFP into MDCK-EGFP-E47 (clone #6) cells are shown in **A** and **B**, respectively. (**C**) The tumorigenic potential of MDCK-EGFP-E47#6 and MDCK-EGFP-E47-shEGFP#N and #P cells was analysed by subcutaneous injection into nude mice. ANOVA analysis: *p<0.05; **p<0.01; ***p<0.001. (**D**) Histology (upper panels) and immunohistochemical analysis for E-cadherin (lower panels) of tumours induced by MDCK-EGFP-E47#6 (left) and MDCK-EGFP-E47-shEGFP#N cells (right). Amplification x20. Insets show amplified fields at x40.

One important and still unresolved question is whether continued expression of E47 is necessary for maintenance or just required for induction of EMT in MDCK cells, as reported for E2-2 factors [32]. Since repeated attempts to knockdown E47 by shE47 in MDCK cells were unsuccessful, we decided to silence E47 expression by shRNA against EGFP in MDCK-EGFP-E47. As a control, MDCK-EGFP cells were used to discard side-off effects of the shEGFP. We first verified that EGFP-shRNA is effectively knocking down EGFP and EGFP-E47 in all analysed clones from MDCK-EGFP-shEGFP and MDCK-EGFP-E47-shEGFP, respectively (Figure 3A, panels g,i,j, and 3B). Control MDCK-EGFP cells after shEGFP remained with an epithelial phenotype, expressing high levels of E-cadherin and other epithelial markers and lacking Id1 expression (Figure 3A, B). In contrast, EGFP-E47 silencing by shEGFP induced a remarkable reversion to an epithelial phenotype in MDCK-EGFP-E47 cells (Figure 3A, panels d,e), associated with re-expression of E-cadherin and its re-localization at cell-cell contacts (Figure 3A and B). Upregulation and membrane re-localization of the epithelial markers beta-catenin and plakoglobin was also observed (Figure 3A and B). Moreover, EGFP-E47 silencing promoted the reorganization of the F-actin cytoskeleton from stress fibers and lamellipodia-like structures to cortical actin filaments (Figure 3A panels x,y, *vs* panel w). Significantly, EGFP-E47 silencing was associated to almost complete suppression of Id1 overexpression in MDCK-EGFP-E47-shEGFP clones (Figure 3B), further supporting the link between expression of E47, EMT and Id1 induction in the MDCK cell system.

The relevance of E47 interference in the tumorigenic properties of MDCK-EGFP-E47 cells was then analysed. EGFP-E47-interfered clones were injected into nude mice, in parallel with control MDCK-EGFP-E47 cells that exhibit similar tumorigenic properties than previously reported for MDCK-E47 cells [28], [29], [31]. All cell lines gave rise to tumours at all injection sites. However, the shEGFP-E47 derived tumours grew at low rate with an overall 50% to 85% reduction of tumour volume compared to the tumours induced by control cells (Figure 3C). Tumours induced by MDCK-EGFP-E47 cells were highly undifferentiated spindle cell tumours, without evidence of epithelial differentiation (Figure 3D, left). In contrast, tumours generated by MDCK-EGFP-E47-shEGFP cells showed areas of glandular differentiation surrounded by undifferentiated spindle cells (Figure 3D, right) or were very small tumours (Figure 3C). Moreover, the MDCK-EGFP-E47-shEGFP xenografts express E-cadherin at differentiated areas in contrast to the expected absence of E-cadherin expression in control MDCK-EGFP-E47 tumours (Figure 3D, lower panels).

These results indicate that sustained E47 expression is required for the maintenance of the full phenotypic characteristics and markers of EMT, including Id1 expression and for aggressive tumorigenic properties of MDCK-E47 expressing cells.

### Id1 expression is required for EMT induced by E47 and cell survival

**Figure 4:**
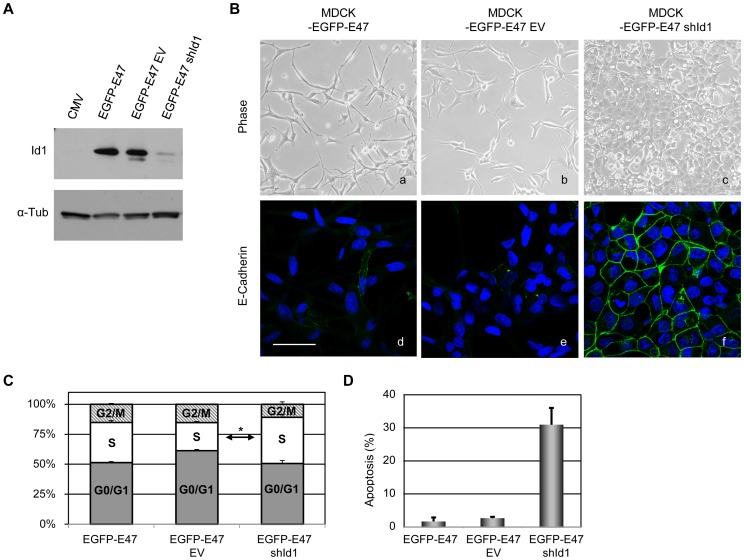
Id1 knockdown in MDCK-EGFP-E47 cells induces a partial MET and impairs cell survival. (**A**) Western blot analysis of Id1 expression in MDCK-EGFP-E47 cells after 48 h infection with shId1 or control lentivirus (EV), compared to non-infected (EGFP-E47) and control MDCK-CMV (CMV) cells; alpha-tubulin was used as loading control. (**B**) Phase contrast (upper panels) and confocal immunofluorescence images of E-cadherin (lower panels) of EGFP-E47-shId1 (right) and EGFP-E47-EV control (middle) cells 48 h after lentiviral infection; non infected MDCK-EGFP-E47 cells (left) are shown as additional control. Nuclei were stained with DAPI; bars, 50 um. (**C**) Cell cycle analysis of the indicated cell lines by propidium iodide staining and FACS analysis. (**D**) Apoptosis rate of the indicated cells determined by the sub-G0 population. Results in **C** and **D** represent the mean +/− s.d. on triplicate samples.

Once established that E47 is required to maintain the EMT phenotype, we decided to study whether EMT also depends on induction of Id1 in MDCK-E47 cells. To answer this point, we knocked down Id1 in MDCK-EGFP-E47 cells by lentiviral infection. Three independent shId1 lentiviral constructs were tested and results obtained with shId1 9032 are shown (Figure 4). It should be noted that the dog genome is only partially annotated and shId1 sequences were selected based on one Id1 sequence with strong human/dog homology. However, only shId1 9032 of the three analysed shId1 lentivirus blocked efficiently endogenous Id1 in dog MCDK cells (data not shown). To discard side-off effects, the action of the selected shId1 construct on MDCK-EGFP-E47 cells was compared with control lentivirus and with non-infected cells. Efficient knockdown of Id1 was detected 48 h after infection (about 90%) with no significant effect detected with the control lentivirus (Figure 4A). After 48 h of infection, Id1 knockdown induced a striking reversion to an epithelial-like phenotype in MDCK-EGFP-E47-shId1 cells (Figure 4A, panel c) compared to non-infected and control infected cells that remain fully mesenchymal (Figure 4B, panels a,b). The phenotypic Mesenchymal to Epithelial (MET)-like reversion was accompanied by strong organization of E-cadherin at cell-cell contacts in MDCK-EGFP-E47-shId1 cells (Figure 4B, panel f) compared to parental and control cells (Figure 4B, panels d,e). Knockdown of Id1 for longer time periods induced a massive cell death; indeed, repeated attempts to obtain stable infections with shId1 lentivirus were unsuccessful. Analysis of the cell cycle indicated no major alterations in MDCK-EGFP-E47-shId1 cells compared to parental cells (Figure 4C). However, a dramatic increase in the sub-G0 population was detected in MDCK-EGFP-E47-shId1 cells indicative of a high apoptotic rate compared to either parental and control cells (Figure 4D).

Together, these results indicate that expression of Id1 is required for maintenance of the mesenchymal phenotype after EMT and cell viability of MDCK-EGFP-E47 cells.

### E47 and ID factors expression in breast cancer

At present, very little is known about the expression of E47 and IDs and their correlation with specific subtypes of human breast carcinomas, although *ID1* expression has been correlated with poor prognosis, metaplastic tumours and lung metastasis in breast carcinomas [20]–[22], [34], [35]. To investigate the biological significance of E47 and ID factors in human breast cancer we analyzed their expression together with markers defining the basal and non-basal subtypes in unsupervised clustering analysis in a series of 97 sporadic N0 breast carcinomas profiled by Vańt Veer *et al*. [36].

**Figure 5:**
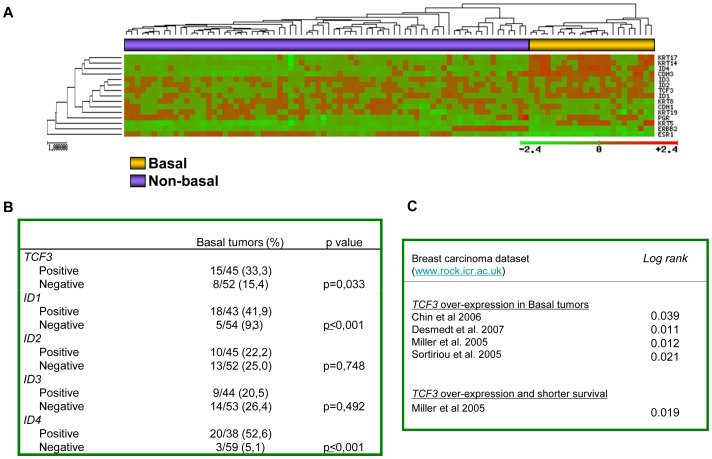
E47 and ID factors are preferentially expressed in basal-like breast carcinomas. (**A**) Unsupervised hierarchical clustering of the vant’Veer dataset (ref. 36) for the expression of the indicated basal and luminal markers, together with *estrogen* (*ESR1*) and *progesterone receptors* (*PGR*) and *ErbB2*, identifies two clusters of basal (yellow) and non-basal (purple) tumours. Increased *TCF3* and *ID1/ID4* transcripts cluster with the basal-like subgroup. (**B**) Association between the expression of *TCF3* and *ID1/ID4* with the basal-like phenotype in the vańt Veer's dataset. To validate the association among specific genes and/or tumour subgroups, each variable was categorized using the percentile 75 value as the cut-off. (**C**) Overexpression of *TCF3* associated to basal-like breast tumours (upper) and to decreased overall survival (bottom) in the indicated public datasets.

As shown in Figure 5A, tumours were sub-classified into two main clusters, indicating that *TCF3* (*E47/E12*) and *ID* genes are differentially expressed among breast carcinomas. Attending to the markers defining tumour subgroups, the right cluster (yellow bar) contained the hormone-receptor-negative tumours; according to the low expression of *ESR*, *PGR*, *ERBB2* and luminal keratins (*KRT8* and *KRT19*) and high expression of basal keratins (*KRT5, KRT14, KRT17*) this subgroup corresponds to the basal-like tumours. The left-handed cluster (purple bar) includes the non-basal tumours (luminal and ErbB2+). In this dataset, positive *TCF3*, *ID1* and *ID4* expression were more frequent (Chi-square test, p = 0.033, p<0.001, and p<0.001, respectively) in the basal-like tumours (Figure 5B). Moreover, *TCF3*, *ID1* and *ID4* expression showed a strong association with the expression of basal markers, such as *CK14*, *CK17*, and *P-cadherin* (Table S1). These results agree with and complement previous studies on ID1 protein expression in metaplastic and triple negative breast cancer tumours [21], [34]. Furthermore, the analysis of *TCF3* expression in several breast carcinomas dataset [37]–[40] showed that *TCF3* upregulation associated to the basal-like phenotype (Figure 5C, upper) and exemplified in dedifferentiated breast carcinoma MDA-MB435 cells (Figure 1D). In addition, *TCF3* upregulation was associated to shorter overall survival in the Miller's series [39] (Figure 5C, bottom). Taken together, these results suggest *TCF3* as a new marker of basal-like breast tumors and support the interplay between E47 and ID factors in this tumour subtype.

## Discussion

Class I bHLH E2A factors (E12/E47) have been previously shown to promote *E-cadherin* repression and to induce EMT and invasive, tumorigenic and angiogenic or fibrotic properties when expressed in epithelial cells [17], [28], [29], [31], [41]. However, the mechanisms underlying *E-cadherin* repression and the specific role of E12/E47 in EMT have not yet been elucidated. We report here that E47 transcriptional repressor binds directly to the *E-cadherin* promoter in MDCK cells stably expressing E47 factor. We also show that continued expression of E47 and Id1 is necessary for maintenance of EMT in the MDCK-EGFP-E47 cell system.

Consistent with the dominant negative action of Ids on bHLH factors [15], [16] we show that Id1 protein efficiently overcome E47-induced repression of *E-cadherin* promoter in transient co-transfection assays in epithelial MDCK cells, similar to previously reported for Id3 [17]. However, overexpressed Id1 protein is unable to overcome E47-dependent repression of *E-cadherin* expression in mesenchymal MDCK-E47 cells; a similar situation was observed for Id2 and Id3 (unpublished data). Indeed, the ChIPs assays show that E47 binds to the endogenous *E-cadherin* promoter of MDCK-EGFP-E47 cells in complexes devoid of Id1. Nonetheless, this situation occurs despite the physical interaction between E47 and Id1 detected in mesenchymal MDCK-EGFP-E47 cells. The E47/Id1 interaction and inability of Id1 to reactivate the *E-cadherin* promoter was further confirmed in E47-expressing basal-like breast carcinoma and melanoma cells. One possible explanation to reconcile these apparently contradictory findings is that post-translational modifications might regulate E47 transcriptional activity, as previously described for site-specific phosphorylation and/or covalent disulfide bond dimerization [42]-[45]; such modifications in a subset of E47 molecules might attenuate their interaction with Id1 and allow E47 binding to the *E-cadherin* promoter in absence of Id1. It is tempting to speculate that this subset of E47 molecules can act as active homodimers promoting *E-cadherin* repression; in fact, our previous *in vitro* band-shift and *in vivo* yeast-one-hybrid analysis indicated that E47 interacts with the mouse *E-cadherin* promoter as a homodimer [29], [33]. Another possibility is the interaction of E47 with tissue-specific class II bHLH factors [15]. One potential candidate is Twist1, another EMT inducer [46]; we tested this hypothesis, but we could not find any functional E47/Twist1 interaction in *E-cadherin* promoter assays (unpublished data) in line with recent data suggesting an indirect role of Twist1 in *E-cadherin* repression [3], [47]. Alternatively, the presence of activating E proteins sequestered by Id1 induction in the presence of E47 could be another option. Although further studies are clearly required to clarify the mechanism, in the present scenario, we favour the hypothesis that a subset of E47 molecules with potential post-translational modifications could be responsible of *E-cadherin* repression likely in homodimeric form.

The present data also provide evidence for a strong link between E47 and Id1 in EMT. Sustained expression of E47 is required to maintain the mesenchymal phenotype after EMT, in contrast to the behaviour of E2-2 factors that act as EMT-inducers but are dispensable for maintenance of EMT [32]. Significantly, the MET induced by E47-silencing in MDCK-EGFP-E47 cells is associated to strong downregulation of Id1. Moreover, Id1 knockdown also induces a MET-like process in MDCK-EGFP-E47 cells, indicating that expression of Id1 is indeed required for maintenance of EMT induced by E47 and, importantly, for cell survival. These results agree with the pleiotropic effect of Id1 on cell proliferation and survival, partly independent of E2A proteins [16], [48], and support the collaboration between E47 and Id1 in EMT beyond *E-cadherin* repression. Interestingly, Id1 is induced by additional EMT-TFs, like Snail1, Snail2 and E2-2A/B [30]-[32], further supporting its participation in maintenance of EMT. Whether the specific action of Id1 in EMT maintenance is dependent of its interaction with other nuclear, cytosolic or membrane proteins remains to be established.

Many studies highlight distinct roles for Id proteins in tumorigenesis, invasiveness and metastasis in epithelial, endothelial and hematopoietic cells [16]. Furthermore, ID1 overexpression has been associated with malignancy, poor clinical outcome or lung metastasis in different tumour types [20]–[22], [25], [34], [35], [49]. Overexpression of E47 in some tumours and cell lines has also been reported [29], [50], [51]. Interestingly, our analysis of *E47* (*TCF3*) and *IDs* expression in the N0 breast carcinoma series profiled by Van’t Veer *et al*. [36] indicates that the expression of *E47* and *ID1* is significantly associated with the basal-like phenotype in sporadic tumours, in agreement with previous observations on ID1 protein expression [21]. Overexpression of *ID4* was also associated with basal-like tumours, in agreement with a recent report on ID4 protein overexpression in triple negative breast tumours (TNBC) [52], and also detected in the vant’ Veer series (Figure 5B). Rrecent studies have highlighted the connection between ID4 induction and downregulation of BRCA1 in TNBCs [53] as well as with p53 mutation in estrogen receptor negative (ER-) breast tumours [54] that might contribute to chemoresistance of p53 mutant breast tumours [55]. Interestingly, E47 has been also reported to contribute to doxorubicin chemoresistance [56]. Upregulation of *Id4* transcripts were also previously observed in MDCK cells overexpressing E47, although to a lower extent than *Id1* [31], supporting also the correlation between *ID4* and *TCF3* upregulation in basal breast tumours. Whether ID1 and ID4 play non-redundant or complementary actions on EMT and/or the basal phenotype and chemoresistance of breast tumours remains to be established in future studies. Importantly, our present study detected *TCF3* upregulation in basal-like tumours in several additional public datasets and associated to poor survival (Figure 5C), reinforcing previous observations indicating that basal-like carcinomas are the most likely candidate breast tumours to suffer EMT processes [57]-[59]. A few pathways and molecules have been so far implicated in the regulation of epithelial plasticity in basal-like tumours, including the Wnt signaling, Forkhead 1 factor FOXC2, and lysyl oxidase-like 2 (LOXL2) [60]–[63]. The evidences here presented suggest that additional genes such as *TCF3* and *ID1* could also mediate EMT in basal-like carcinomas. Furthermore, our previous observation that upregulation of *TCF8* (ZEB1) occurs in metastatic and more undifferentiated breast tumours of the Vańt Veer's series [64] suggest a potential link between E47/ID1 or ID4 and TCF8 expression in undifferentiated basal-like tumours. Although further studies are required, our present observations on the *E47* and *ID1* expression profile in the N0 breast carcinoma series provide biological basis to the interplay between E47 and ID1 here described.

## Materials and Methods

### Plasmid constructs

Enhanced green fluorescent protein EGFP-E47 construct was generated by PCR using the full-length mouse E47 cDNA as template [29] with the following primers (restriction sites indicated in bold): 5′-**CTCGAG**GGATGATGAACCAGTCTCA-3′ (forward) and 5′-**GAATTC**GGCTCACAGGTGCCCGGCTGGGT -3′ (reverse). The PCR product was cloned in the pEGFP-C1 vector (Clontech, Mountain View; CA). Mouse Id1 construct (pcDNA3-Id1) was provided by Dr. M. Saitoh (Tokio University, Japan).

### Cell culture and gain of function studies

MDCK-II (Madin Darby canine kidney type II) and human HEK293T (human embryonic kidney 293 transformed with T-antigen), MDA-MB435S whose origin has been firmly established as dedifferentiated breast carcinoma [65], [66] and melanoma A375P cells were obtained from the American Type Cell Culture (ATCC) (LGC Standards-SLU; Barcelona, Spain) and cultured as previously described [29]. Stable transfectants were obtained from parental MDCK-II (Madin Darby canine kidney type II) cells (obtained from the ATCC) (LGC Standards-SLU; Barcelona, Spain) after transfection with pEGFP-C1-E47, or the corresponding control vector, using Lipofectamine (Invitrogen; Carlsbad, CA). Cells were grown as previously described [67] in the presence of G418 (400 µg/ml; Gibco; Madrid, Spain) for 3–4 weeks and individual clones isolated with cloning rings. At least ten independent clones were isolated and results from two representative clones from each setting are shown. The origin and characterization of MDCK-CMV and MDCK-E47 cells has been previously described [29], [67].

### RNA interference

The generation of shRNA containing specific oligonucleotide sequences against EGFP cloned into the pSuperior-Puro vector (Oligoengine; Seattle, WA) was previously described [30]. MDCK-EGFP and MDCK-EGFP-E47 (clone #6) cells were transfected with shEGFP vector using Lipofectamine 2000 (Invitrogen) and sorted using a FACSVantage SE (Becton Dickinson; Madrid, Spain) to obtain populations with more than 90% EGFP-negative cells. At least ten clones were isolated from each pool by limited dilution and results from two to five independent clones from each setting are shown.

### Lentiviral infection

Id1 knockdown was performed by lentiviral infection with specific shRNA vectors (TRCN0000019032; Sigma-Aldrich; St Louis, MO). Briefly, HEK293T cells were transfected with a mix containing VSV-G envelope and ΔR 8.91 packaging genes and either control vector (pLKO: EV) or Id1 specific shRNA using Lipofectamine 2000 and incubated for 48 h. MDCK-EGFP-E47 cells were infected with the lentiviral supernatant with polybrene (8 ng/ml) during 48 h.

### *E-cadherin* promoter assays

The activity of the mouse *E-cadherin* (*Cdh1*) promoter on HEK293T, MDCK and MDCK-derived clones, MDA-MB435S and A375P cells was determined as described [67]. Mutants in the E-pal and/or E3-box were previously described [32]. Statistical analyses were performed by the two paired t-Student’s test.

### Northern Blotting

15 to 30 ug of total RNA from the different cell lines were analysed in Northern blot using a specific P^32^-cDNA *Cdh1* probe as described [30].

### Cell extracts, Western blot analysis and immunoprecipitations

Whole cell extracts, lysates, immunoprecipitation conditions and Western blot analyses were performed as described [11], [12], [67]. The primary and secondary antibodies used are described in Table S2.

### Chromatin immunoprecipitation (ChIP) and Sequential ChIP assays

ChIP assays were performed in MDCK-EGFP-E47 (clone #6) cells, using formaldehyde before sonication, as described [11], [32]. For detection of interaction between the tagged factors and the endogenous *Cdh1* promoter, an anti-EGFP antibody (Molecular Probes; Grand Island, NY) or unspecific rabbit IgG (Jackson ImmunoResearch Laboratories; Suffolk, UK) were used. A 215 bp fragment of the dog *Cdh1* promoter (–85/+130) was amplified using the primers and amplification conditions previously described [32]. For re-ChIP assays, the immune complexes were harvested with elution buffer (TE 1X, 2% SDS, 15 mM DTT and protease inhibitors), diluted 1∶10 with dilution buffer (10 mM Tris-HCl [pH 8], 150 mM NaCl, 1 mM EDTA, 0.01% SDS, 0.5% Nonidet P-40 and protease inhibitors), and the steps of the ChIP assay repeated with a second round of immunoprecipitation using anti-EGFP (Molecular Probes) or anti-Id1 (Santa Cruz; California, CA) antibodies.

### Cell cycle and apoptosis assays

Cell cycle analysis was performed by propidium iodide staining. Semi-confluent cells were trypsinized and washed twice with PBS, fixed with cold 70% ethanol, washed, resuspended in PBS with 10 ug/ml of propidium iodide, and analysed in a FACScanI (Becton Dickinson) using a ModFIT LT v3.2 software. Assays were performed on triplicate samples.

### Immunofluorescence

Immunofluorescence analysis and F-actin stain was performed as described [67]. The primary and secondary antibodies used are described in Table S2. Preparations were visualized using a Zeiss Axiophot or a Leica confocal TCSSP2 microscope.

### Induction of xenografted tumours and histological studies

Subconfluent cells were trypsinised, washed and resuspended at 1×10^7^ cells/ml in PBS. 1×10^6^ cells were injected into the flanks of 8-week old male BALB/c nude mice (Charles River; Wilmington, MA). Tumours were measured every 3–4 days using a calliper by determination of the two orthogonal external diameters; statistical comparisons were made by ANOVA analysis. Mice were sacrificed when the tumours reach 0.1 cm^3^ size; tumours were surgically excised and processed for histology as described [67]. Mice were housed and maintained under specific pathogen-free conditions and used in accordance with institutional guidelines and approved by the Use Committee for Animal Care. The protocol was approved by the Committee on the Ethics of the University Autonomous of Madrid (UAM. Permit Number: CEI-25-587) and Spanish National Research Council (CSIC), according with present Spanish law (R.D. 1201/2005, 10th October, BOE 21st October 2005). All animals were monitored for ill effects and sacrificed at 32 days post-injection; all efforts were made to minimize suffering according to UAM/CSIC guidelines. A minimum of 4 tumours from each cell line were generated and analysed by histology and immunohistochemistry.

### Human breast cancer expression data set and statistical analysis

Microarray and clinical data from 97 sporadic breast tumours, reported by van’t Veer et *al.* [36] were used. The sporadic samples included primary invasive carcinoma <5 cm (T1 or T2), with no axillary lymph node metastases (N0). To classify the breast samples according to basal and non-basal (luminal and ErbB22+) phenotypes, we extracted the log ratio values of *ESR1* (*estrogen receptor*), *PGR* (*progesterone receptor*), *ERBB2*, and luminal (*CDH1*, *KRT8*, and *KRT19*) and basal markers (*CDH3*, *KRT5*, *KRT14*, and *KRT17)* present in the Vańt Veer dataset; in addition, we select the *TCF3 (E47)* and *ID* (*ID1*–*4*) factors expression. Hierarchical cluster analysis was performed assuming euclidean distances between genes. For significance associations Yates correction or Fisher’s exact test were used. To evaluate *TCF3* association to breast carcinoma subtypes, several independent dataset of carcinoma series were also analysed [37]–[40]. The clinical data were obtained from the ICR database (www.rock.icr.ac.uk). *TCF3* expression values were categorized using upper quartile (25% against rest).

## Supporting Information

Figure S1 **Stable expression of EGFP-E47 in MDCK cells triggers a complete EMT.** (**A**) Phase contrast (a–c) and immunofluorescence (d–l) images of the indicated cell lines for EGFP (d), EGFP-E47 (e,f), E-cadherin (g–i) and vimentin (j–l). Nuclei were stained with DAPI (g–l). Bars, 60 mm (a–c); 25 mm (d–l). (**B** and **C**). Western blot (**B**) and Northern blot analysis (**C**) analyses of the indicated markers in E47 expressing cells and controls. alpha-tubulin and GAPDH were used as loading controls.
(TIF)

Figure S2 ***E-cadherin*** **promoter activity in A375P (left) and MDA-435S (right) cells transiently cotransfected with 200 ng of the proximal mouse** ***E-cadherin*** **promoter and increasing amounts of Id1 and 10 ng of CMV-beta-gal.** RLU is normalized to activity detected in the presence of pcDNA3. Results represent the mean +/- s.d. of three independent experiments performed on triplicate samples.
(TIF)

Table S1 **Association of TCF3 positive expression with basal and luminal markers in N0 breast tumours.** The categorical expression of progesterone receptor (PGR), estrogen receptor (ESR1), ERBB2, and basal and luminal markers in the van’t Veer’s dataset (ref. 38) is shown.
(DOC)

Table S2 **Antibodies used in Western-blot, immunofluorescence immunoprecipitation and IHQ assays.**
(DOC)
