# Supplementary figures and images for: E47 and Id1 Interplay in Epithelial-Mesenchymal Transition
Source: PLoS One. 2013 Mar 26;8(3):e59948. doi: 10.1371/journal.pone.0059948 (PMC3608585; doi:10.1371/journal.pone.0059948)

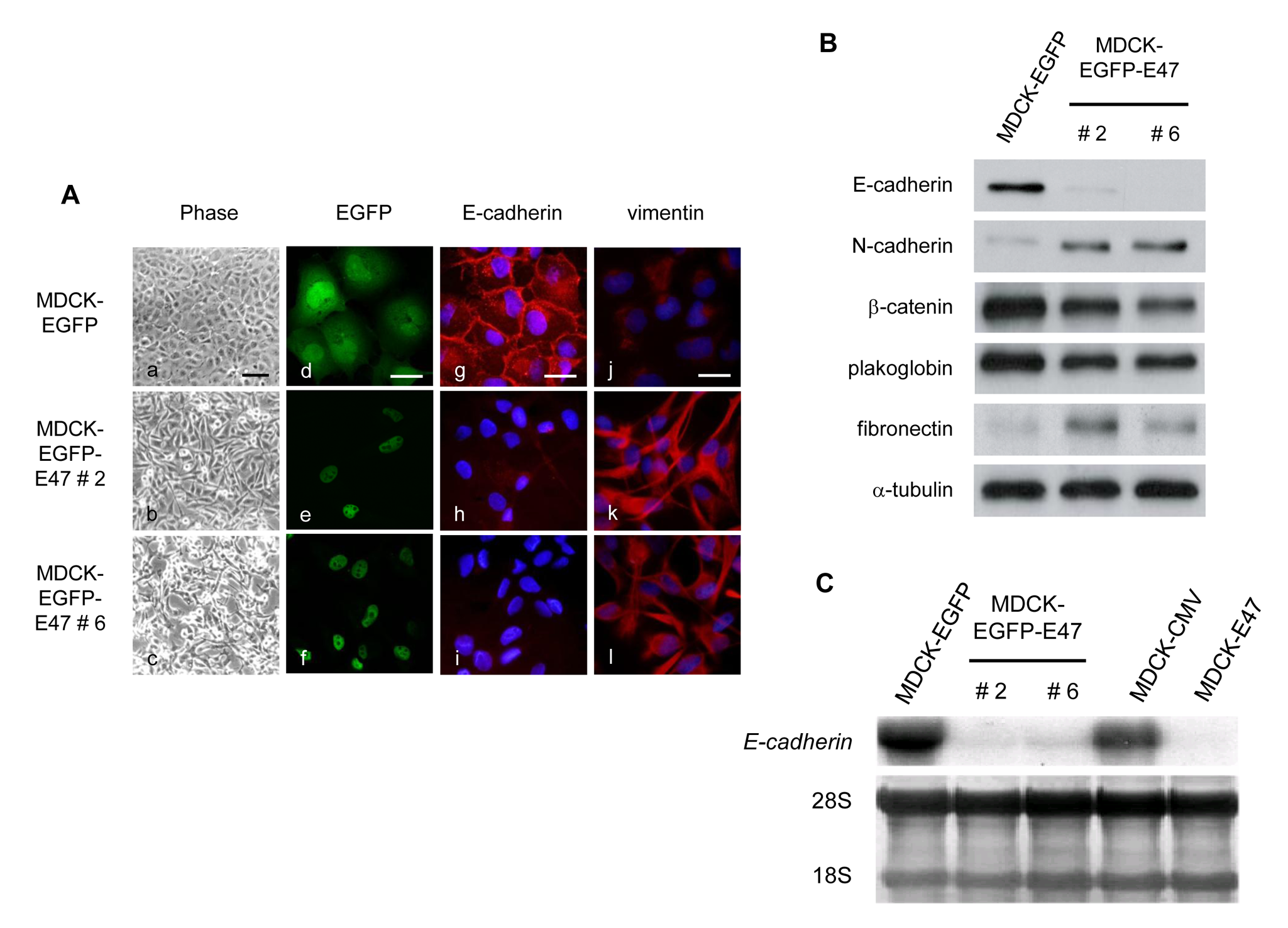

Supplement: Figure S1 — Stable expression of EGFP-E47 in MDCK cells triggers a complete EMT. (A) Phase contrast (a–c) and immunofluorescence (d–l) images of the indicated cell lines for EGFP (d), EGFP-E47 (e,f), E-cadherin (g–i) and vimentin (j–l). Nuclei were stained with DAPI (g–l). Bars, 60 mm (a–c); 25 mm (d–l). (B and C). Western blot (B) and Northern blot analysis (C) analyses of the indicated markers in E47 expressing cells and controls. alpha-tubulin and GAPDH were used as loading controls. (TIF) [file pone.0059948.s001.tif]

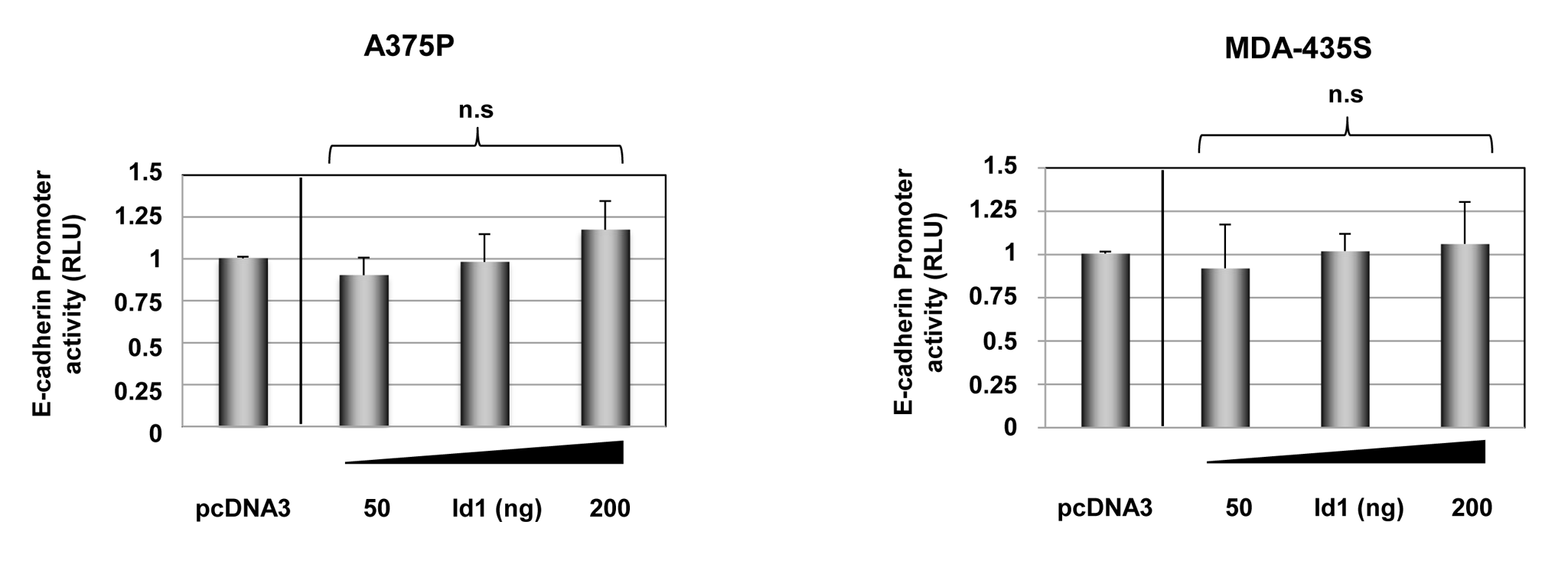

Supplement: Figure S2 — E-cadherin promoter activity in A375P (left) and MDA-435S (right) cells transiently cotransfected with 200 ng of the proximal mouse E-cadherin promoter and increasing amounts of Id1 and 10 ng of CMV-beta-gal. RLU is normalized to activity detected in the presence of pcDNA3. Results represent the mean +/- s.d. of three independent experiments performed on triplicate samples. (TIF) [file pone.0059948.s002.tif]
