# Supplementary material for: E47 and Id1 Interplay in Epithelial-Mesenchymal Transition
Source: PLoS One. 2013 Mar 26;8(3):e59948. doi: 10.1371/journal.pone.0059948 (PMC3608585; doi:10.1371/journal.pone.0059948)
Supplement: Table S1 — Association of TCF3 positive expression with basal and luminal markers in N0 breast tumours. The categorical expression of progesterone receptor (PGR), estrogen receptor (ESR1), ERBB2, and basal and luminal markers in the van’t Veer’s dataset (ref. 38) is shown. (DOC) [file pone.0059948.s003.doc]

**Table S1.** Association of *TCF3* positive expression with basal and luminal markers in N0 breast tumours.

|  | **TCF3 positive (%)** | **ID1 positive (%)** | **ID2 positive (%)** | **ID3 positive (%)** | **ID4 positive (%)** |
| --- | --- | --- | --- | --- | --- |
| **ESR1**  **Positive**  **Negative** | 24/61 (39,3)  21/36 (58,3)  p=0,055 | 22/61 (36,1)  21/36 (58,3)  **p=0,033** | 27/61 (44,3)  18/36 (50,0)  p=0,584 | 29/61 (47,5)  15/36 (41,7)  p=0,575 | 16/61 (26.2)  22/36 (61.1)  **p=0.001** |
| **PGR**  **Positive**  **Negative** | 20/48 (41,7)  25/49 (51,0)  p=0,356 | 23/48 (47,9)  20/49 (40,8)  p=0,482 | 18/48 (37,5)  27/49 (55,1)  p=0,082 | 22/48 (45,8)  22/49 (44,9)  p=0,926 | 17/48 (35.4)  21/49 (42.9)  p=0.453 |
| **ERBB2**  **Positive**  **Negative** | 15/30 (50,0)  30/67 (44,8)  p=0,633 | 9/30 (30,0)  34/67 (50,7)  p=0,057 | 14/30 (46,7)  31/67 (46,3)  p=0,971 | 14/30 (46,7)  30/67 (44,8)  p=0,863 | 8/30 (26.7)  30/67 (44.8)  p=0.091 |
| **KRT5**  **Positive**  **Negative** | 28/4 (63,6)  17/53 (32,1)  p=0,002 | 28/44 (63,6)  15/53 (28,3)  **p<0,001** | 22/44 (50,0)  23/53 (43,4)  p=0,516 | 19/44 (43,2)  25/53 (47,2)  p=0,694 | 27/44 (61.4)  11/53 (20.8)  **p<0.001** |
| **KRT14**  **Positive**  **Negative** | 23/33 (69,7)  22/64 (34,4)  p=0,001 | 20/33 (60,6)  23/64 (35,9)  p=0,020 | 15/33 (45,5)  30/64 (46,9)  p=0,894 | 14/33 (42,4)  30/64 (46,9)  p=0,677 | 20/33 (60.6)  18/64 (28.1)  p=0.002 |
| **KRT17**  **Positive**  **Negative** | 27/43 (62,8)  18/54 (33,3)  p=0,004 | 25/43 (58,1)  18/54 (33,3)  p=0,015 | 22/43 (51,2)  23/54 (42,6)  p=0,400 | 17/43 (39,5)  27/54 (50,0)  p=0,304 | 22/43 (51.2)  16/54 (29.6)  p=0.031 |
| **KRT8**  **Positive**  **Negative** | 21/49 (42,9)  24/48 (50,0)  p=0,481 | 16/49 (32,7)  27/48 (56,3)  p=0,019 | 22/49 (44,9)  23/48 (47,9)  p=0,766 | 24/49 (49,0)  20/48 (41,7)  p=0,469 | 10/49 (20.4)  28/48 (58.3)  p<0.001 |
| **KRT19**  **Positive**  **Negative** | 26/58 (44,8)  19/39 (48,7)  p=0,706 | 23/58 (39,7)  20/39 (51,3)  p=0,258 | 28/58 (48,3)  17/39 (43,6)  p=0,650 | 27/58 (46,6)  17/39 (43,6)  p=0,774 | 19/59 (32.8)  19/39 (48.7)  p=0.114 |
| **P-cadherin**  **Positive**  **Negative** | 27/41 (65,9)  18/56 (32,1)  p=0,001 | 21/41 (51,2)  22/56 (39,3) p=0,243 | 18/41 (43,9)  27/56 (48,2)  p=0,674 | 16/41 (39,0)  28/56 (50,0)  p=0,283 | 22/41 (53.7)  16/56 (28.6)  p=0.012 |
| **E-cadherin**  **Positive**  **Negative** | 20/51 (39,2)  25/46 (54,3)  p=0,136 | 18/51 (35,3)  25/46 (54,3)  p=0,059 | 24/51 (47,1)  21/46 (45,7)  p=0,890 | 24/51 (47,1)  20/45 (43,5)  p=0,724 | 15/51 (29.4)  23/46 (50.0)  p=0.038 |
| **TP63**  **Positive**  **Negative** | 14/35 (40,0)  31/61 (50,8)  p=0,307 | 19/35 (54,3)  24/61 (39,3)  p=0,156 | 13/35 (37,1)  32/61 (52,5)  p=0,148 | 14/35 (40,0)  30/61 (49,2)  p=0,385 | 11/35 (39.6)  27/61 (44.3)  p=0.216 |
