# Supplementary material for: E47 and Id1 Interplay in Epithelial-Mesenchymal Transition
Source: PLoS One. 2013 Mar 26;8(3):e59948. doi: 10.1371/journal.pone.0059948 (PMC3608585; doi:10.1371/journal.pone.0059948)
Supplement: Table S2 — Antibodies used in Western-blot, immunofluorescence immunoprecipitation and IHQ assays. (DOC) [file pone.0059948.s004.doc]

Tabla S2. Antibodies used in Western-blot, immunofluorescence, immunoprecipitation and IHQ assays.

| **Antibody** | **Host** | **Application** | | | **Source** |
| --- | --- | --- | --- | --- | --- |
|  |  | **WB** | **IF** | **IP IHQ** |  |
| **Primary antibodies:** |  |  |  |  |  |
| E-cadherin (ECCD-2) | Rat | 1:100 | 1:50 | 1:100 | M.Takeihi, Kyoto University, Japan |
| N-cadherin (3B9) | Mouse | 1:200 | 1:500 |  | Zymed Laboratories |
| fibronectin | Rabbit | 1:1,000 | 1:200 |  | CHEMICON International |
| vimentin (V9) | Mouse | 1:500 | 1:100 |  | Dako |
| β-catenin (14) | Mouse | 1:500 | 1:200 |  | BD Transduction |
| plakoglobin (15) | Mouse | 1:500 | 1:200 |  | BD Transduction |
| α-tubulin (DM1A) | Mouse | 1:10,000 | - |  | Sigma-Aldrich |
| E2A | Rabbit | 1:500 | - |  | A.T.,Look, Dana-Farber Cancer Institute, Boston, USA |
| ID1 (C-20): sc-488 | Rabbit | 1:500 | 1:50 | 1:500 | Santa Cruz Biotechnology |
| ID3 (C-20): sc-490 | Rabbit | 1:250 | - |  | Santa Cruz Biotechnology |
| EGFP | Rabbit | 1:5,000 | - |  | Sigma-Aldrich |
| EGFP | Rabbit | - | 1:200 |  | Molecular Probes |
| **Secondary antibodies:** |  |  |  |  |  |
| rabbit-HRP | Goat | 1:4,000 |  |  | Nordic |
| rat-HRP | Sheep | 1:1,000 |  |  | Amersham |
| rat-HRP | Goat | 1:10,000 |  |  | Nordic |
| rat Alexa 488/594/647 | Goat |  | 1:1,000 |  | Molecular probes |
| rat Alexa 488/594/647 | Goat |  | 1:1,000 |  | Molecular probes |
| rat Alexa 488/594/647 | Goat |  | 1:1,000 |  | Molecular probes |
|  |  |  |  |  |  |
|  |  |  |  |  |  |
|  |  |  |  |  |  |

HRP = horseradish peroxidase; WB = Western blot; IF = Immunofluorescence;

IP = Immunoprecipitation; IHQ = Immunohistochemistry
